# Diagnose und Management der Osteoporose bei Diabetes mellitus (Update 2026)

**DOI:** 10.1007/s00508-025-02664-x

**Published:** 2026-04-30

**Authors:** Christian Muschitz, Alexandra Kautzky-Willer, Martina Rauner, Yvonne Winhöfer-Stöckl, Judith Haschka

**Affiliations:** 1 II. Medizinische Abteilung, Krankenhaus der Barmherzigen Schwestern Wien, Wien, Österreich; 2 Universitätsklinik für Innere Medizin III, Klinische Abteilung für Endokrinologie und Stoffwechsel, Wien, Österreich; 3 Bone Lab Dresden, Medizinische Klinik und Poliklinik III, Medizinische Fakultät, Technische Universität Dresden, Dresden, Deutschland; 4 Innere Medizin & Rheumatologie, healthPi Medical Center Wien, Wien, Österreich

**Keywords:** Diabetes, Osteoporose, Fraktur, Medikation, Diabetes-vermittelte Knochenerkrankung, Diabetes, Osteoporosis, Fracture Risk, Medication, Diabetes Related Bone Disease

## Abstract

Diabetes mellitus und Osteoporose zählen zu den häufigsten chronischen Erkrankungen und kommen deshalb beide häufig in ein und demselben Individuum vor. Da die Prävalenz beider mit steigendem Alter zunimmt, wird in Anbetracht der Altersstruktur unserer Bevölkerung deren Häufigkeit zunehmen. Patienten mit Diabetes haben ein erhöhtes Risiko für Fragilitätsfrakturen. Die Pathophysiologie ist unklar und vermutlich multifaktoriell. Longitudinale Studien haben den Nachweis erbracht, dass das Fracture Risk Assessment Tool (FRAX) und die Knochendichte (BMD) mittels DXA-(T-Score)-Messungen und einem eventuell vorhandenen Trabecular Bone Score (TBS) das individuelle Frakturrisiko vorhersagen können. Hierfür muss allerdings eine Adjustierung vorgenommen werden, um das Risiko nicht zu unterschätzen. Es gibt derzeit aus osteologischer Sicht noch nicht den optimalen Ansatz, da es keine Studien mit rein diabetischen Patienten und Osteoporose gibt. Patienten mit Diabetes mellitus und einem erhöhten Frakturrisiko sollten genauso wie Patienten ohne Diabetes und einem erhöhten Frakturrisiko behandelt werden. Der Vitamin-D-Spiegel sollte auf jeden Fall immer optimiert werden, und auf eine ausreichende Kalziumaufnahme (vorzugsweise durch die Nahrung) ist zu achten. Bei der Wahl der antihyperglykämischen Therapie sollten Substanzen mit nachgewiesenem negativem Effekt auf den Knochen weggelassen werden. Bei Vorliegen einer Fragilitätsfraktur ist auf jeden Fall – unabhängig von allen vorliegenden Befunden – eine langfristige spezifische osteologische Therapie indiziert. Zur Prävention von Fragilitätsfrakturen sind antiresorptive Medikamente die erste Wahl, entsprechend den nationalen Erstattungskriterien auch anabole Medikamente. Das Therapiemonitoring soll im Einklang mit der nationalen Osteoporose Leitlinie (Neufassung Herbst 2024) erfolgen.

## Abstract (en)

Diabetes mellitus and osteoporosis are among the most frequent chronic diseases and therefore often occur in one and the same individual. As the prevalence of both diseases increases with age, in view of the age structure of our population their frequency will increase. Patients with diabetes have an elevated risk of fragility fractures. The pathophysiology is unclear and presumably multifactorial. Longitudinal studies have shown that the Fracture Risk Assessment Tool (FRAX) and the bone mineral density (BMD) measured by dual energy X‑ray absorptiometry (DXA, T‑score) and a possibly present trabecular bone score (TBS) can predict the individual risk of fractures; however, an adjustment must be made in order not to underestimate the risk. From an osteological perspective an optimal approach is not yet available as there are no studies with patients who have only diabetes and osteoporosis. Patients with diabetes mellitus and an elevated risk of fractures should be treated in exactly the same way as patients with an increased risk of fractures but without diabetes. The vitamin D level should in every case always be optimized and a sufficient calcium intake should be ensured (preferably via the nutrition). In the selection of the antihyperglycemic treatment, substances with a proven negative effect on bones should be omitted. In the case of a fragility fracture a long-term specific osteological treatment is indicated, independent of all available findings. Antiresorptive medications are the first choice for prevention of fragility fractures and also anabolic drugs according to national reimbursement criteria. Monitoring of treatment should be carried out in compliance with the national osteoporosis guidelines (revised version Autumn 2024).

## Epidemiologie des Diabetes mellitus und osteoporotische Fragilitätsfrakturen

Diabetes mellitus und Osteoporose zählen zu den häufigsten chronischen Erkrankungen und kommen deshalb beide häufig in ein und demselben Individuum vor, weshalb davon ausgegangen wird, dass sie in Zusammenhang stehen. Da die Prävalenz beider mit steigendem Alter zunimmt, wird in Anbetracht der Altersstruktur unserer Bevölkerung deren Häufigkeit zunehmen.

Rezente Metaanalysen mit rund 140.000 Patientinnen und Patienten mit Typ-1-Diabetes (T1DM) zeigen ein gepooltes relatives Risiko (RR) für eine Fraktur von 3,16, für eine Hüftfraktur von 3,78 und für eine vertebrale Fraktur von 2,88. Das RR einer hüftgelenksnahen Fraktur bei Frauen mit T1DM im Vergleich zu Frauen ohne Diabetes beträgt 5,19 [1]. Das Frakturrisiko steigt mit zunehmendem Lebensalter an, speziell Hüftfrakturen treten bei T1DM etwa 10 bis 15 Jahre früher auf [2].

Bei Typ-2-Diabetes (T2DM) weisen populationsbasierte Daten von rund 33.000 Patientinnen und Patienten den T2DM als stärksten Prädiktor für niedrig-traumatische (= osteoporotische) Frakturen bei Männern (RR 2,38) und bei Frauen (RR 1,87) aus [3]. Mit einer durchschnittlichen Odd Ratio (OR) für Frakturen von 1,5 ist der T2DM nur für rund 4 % aller osteoporotischen Frakturen ursächlich in Zusammenhang zu bringen. Dem gegenüber ist allerdings die global steigende Inzidenz von Patientinnen und Patienten mit T2DM (rund 425 Mio. sowie rund 320 Mio. mit einer gestörten Glukosetoleranz) gegenüberzustellen. Zusätzlich zu diesem direkten Risiko kommen noch weitere klinische Risikofaktoren („clinical risk factors“ [CRF]), die mit einem Diabetes einhergehen (z. B. multiple Stürze, Neuro- und Retinopathie etc.) und das individuelle Frakturrisiko zusätzlich erhöhen [4].

## Diabetes assoziierte Risikofaktoren für Frakturen

Diabetes per se ist ein klinischer Risikofaktor für ein erhöhtes Frakturrisiko. Bei T2DM spielen das Alter und die Dauer des Diabetes eine wichtige Rolle. Sowohl bei Frauen als auch bei Männern > 40 Jahren ist T2DM ein unabhängiger Risikofaktor für sämtliche osteoporotische Frakturen (Hazard Ratio [HR] 1,32). Das Alter beeinflusst das Risiko dahin gehend, dass jüngere Patienten ein höheres Risiko für Hüftfrakturen haben (HR Alter < 60 Jahre: 4,67; HR Alter 60 bis 69 Jahre: 2,68; HR Alter 70 bis 79 Jahre: 1,57; HR Alter > 80 Jahre: 1,42) [5]. Entscheidend ist weiters die Dauer der Erkrankung. In den ersten 5 Jahren der Erkrankung kommt es zu keiner Erhöhung des relativen Risikos (ein protektiver Effekt vermehrter Fettmasse wird diskutiert), das Risiko folgt allerdings einem biphasischen Verlauf mit einem zweiten Gipfel jenseits von 10 Jahren Erkrankungsdauer (HR 1,47) [6].

Die glykämische Kontrolle ist wichtig für die Beurteilung des individuellen Frakturrisikos. Ein HbA_1c_ > 7 % führt zu einem raschen Anstieg des Risikos mit einer erhöhten Mortalität nach Frakturen [7]. Zusätzlich hat eine schlechte glykämische Kontrolle einen negativen Einfluss auf die Mikroarchitektur des Knochens mit mikrovaskulären Komplikationen in diesem Organsystem [8].

## Einfluss der Behandlung des Diabetes auf das Frakturrisiko

Das Verhältnis zwischen Diabetes und Knochenfragilität und die Identifizierung jener Patientinnen und Patienten mit einem erhöhten Risiko für Frakturen wird zusätzlich durch die Eigenschaften antidiabetogener Medikamente auf das Skelett beeinflusst (Tab. 1). Obwohl es keine einzige prospektive Studie mit einem primären Studienziel in Bezug auf Therapie des Diabetes und Knochenfragilität gibt, zeigen Daten aus epidemiologischen und Beobachtungsstudien ein heterogenes Muster von teilweise positiven, aber auch negativen Effekten auf den Knochenstoffwechsel.

**Tab. 1:** Einfluss antidiabetogener Medikamente auf BMD („bone mineral density“, Knochendichte) und Frakturrisiko in T2DM

Antidiabetogene Medikation	BMD	Frakturrisiko
*Metformin*	↔/↑	↓/↔
*Sulfonylharnstoffe*	KD	↔/↑
*Thiazolidinedione*	↓↓/↔	↑↑/↔
*Insulin*	↔	↑
*Inkretin-Mimetika*		
DPP-4-Inhibitoren	↔	↓/↔
GLP-1-Analoga	↑/↔	↔
*SGLT-2-Inhibitoren*	↔	↔
Canagliflozin	↔	↑ (?)

↑ Verbesserung/Erhöhung, ↓ Verminderung, ↔ unverändert

*KD* keine Daten, *DPP4* Dipeptidyl-Peptidase-4-Inhibitor, *GLP‑1* Glucagon-like-peptide-1-Analoga, *SGLT‑2* Natrium-Glukose-Cotransporter 2

### Lebensstilfaktoren

Eine Veränderung des Lebensstils ist – nicht nur bei der Erkrankung Diabetes – eine der Säulen der nichtmedikamentösen Therapie. Grundsätzlich ist ein Gewichtsverlust, sofern keine Gegenmaßnahmen gesetzt werden, immer mit dem Verlust von Muskel- und Knochenmasse verbunden. Sarkopenie und sarkopene Adipositas sind Risikofaktoren für Stürze und Gebrechlichkeit, daher ist immer auf eine ausreichende alimentäre Zufuhr von Proteinen und progressives Widerstandstraining zu achten.

Körperliche Aktivität während einer gezielten Gewichtsabnahme verbessert die Lebensqualität und senkt gleichzeitig zirkulierende Sclerostin-Spiegel, unabhängig vom Alter der Patienten [9].

Andere nichtpharmakologische Maßnahmen sind – wie bei vielen anderen Erkrankungen – die Vermeidung von Nikotin und übermäßigem Alkoholgenuss (Abb. 3).

Ein Vitamin-D-Mangel ist sowohl beim T1DM als auch beim T2DM mit hoher Prävalenz vorhanden. Obwohl der direkte Beweis für die Wirksamkeit eines optimierten Vitamin-D-Spiegels bei Adipositas bzw. Diabetes und/oder Insulinresistenz noch nicht in Studien als primärer Endpunkt nachgewiesen wurde, ist doch davon auszugehen, dass Patienten mit Diabetes ähnlich wie nichtdiabetische Kollektive davon profitieren. Ein adäquater Vitamin-D-Spiegel und eine suffiziente Aufnahme von Kalzium (vorzugsweise über die Nahrung) sind daher eine Grundvoraussetzung – auch im Hinblick auf die Prävention eines sekundären Hyperparathyreoidismus. Möglicherweise sind anfänglich höhere Einzeldosen von Cholecalciferol notwendig, um einen suffizienten Spiegel zu erreichen [10]. Eine Supplementation hat allerdings keinen Schutz vor Frakturen, Sturz oder klinisch relevante Effekte auf die Knochendichte gezeigt [11].

### Glykämische Kontrolle

Bei Patienten mit Diabetes besteht zusätzlich eine Fallneigung, welche wahrscheinlich zum erhöhten Frakturrisiko beiträgt. Die periphere Neuropathie, die Retinopathie mit Visusverschlechterung, vermehrte Stürze in der Anamnese, die Tendenz zu hypoglykämen Episoden, die Hypo- oder Hypertension bzw. die autonome Neuropathie sind hier beispielhaft zu nennen.

Eine eng eingestellte glykämische Kontrolle (HbA_1c_ < 7 %) verringert das Frakturrisiko bei Diabetes, vor allem bei älteren Patienten. Allerdings sind sowohl die Hypoglykämie als auch die Hyperglykämie mit einem erhöhten Risiko für Fragilitätsfrakturen assoziiert, wahrscheinlich durch unterschiedliche Mechanismen [12]. Vor allem bei älteren Patienten mit Diabetes wird daher – um das Risiko für hypoglykäme Episoden zu vermeiden – eine weniger stringente Einstellung des Diabetes empfohlen, um das Sturzrisiko zu senken (EASD/ADA Guidelines) [13].

### Effekte antihyperglykämischer Therapie auf den Knochen

#### Metformin

In-vitro-Studien zeigen einen positiven Effekt von Metformin auf die RUNX2(„Runt-related transcription factor 2“)-Expression mit einer vermehrten Aktivierung von Osteoblasten. In Mäusen und Ratten führt die Verabreichung von Metformin zu einer verbesserten Frakturheilung. Zudem kann es dem Knochenverlust in Modellen der Östrogendefizienz bzw. einer ketogenen Diät entgegenwirken. Klinische Daten im humanen und Tiermodell bestätigen einen neutralen bis positiven osteogenen Effekt in Bezug auf Frakturen und bei ketogener Ernährung, sodass diese Medikation in Bezug auf die Knochenqualität als sicher zu werten ist [14–16].

#### Sulfonylharnstoffe

Obwohl In-vitro-Untersuchungen keinen direkten Effekt von Sulfonylharnstoffen auf das Organsystem Knochen nachweisen konnten, zeigen epidemiologische Daten ein erhöhtes Frakturrisiko unter dieser Medikation. Die Hypothese ist ein indirekt erhöhtes Sturzrisiko bedingt durch Hypoglykämien in dieser Substanzgruppe [14]. Hingegen zeigte sich ein neutraler Effekt auf die BMD in kleinen Patientenkollektiven [17, 18].

#### Thiazolidinedione

Sowohl In-vitro- als auch klinische Studien haben bei Rosiglitazon und Pioglitazon einen Verlust von Knochenstruktur nachgewiesen. Thiazolidinedione interagieren mit dem „peroxisome proliferator-activated receptor“ (PPAR)γ, was zu einer Dysbalance zugunsten der Differenzierung von Adipozyten und zulasten der Differenzierung von Osteoblasten führt. Die klinische Konsequenz sind Frakturen. Eine rezente Metaanalyse bestätigt den negativen Einfluss von Pioglitazon beim weiblichen Geschlecht, aber auch für das männliche Geschlecht liegen ähnliche Ergebnisse vor [19]. Es wird daher nach derzeitigem Wissenstand empfohlen, bei postmenopausalen Frauen und auch bei Männern mit einem Risikoprofil für Fragilitätsfrakturen, diese Substanzgruppe eher nicht einzusetzen [20, 21].

#### Insulin

In Observationsstudien wurde häufig ein erhöhtes Frakturrisiko für Patienten unter dieser Therapie beschrieben. Patientinnen und Patienten unter einer Insulintherapie bzw. unter einer Therapie mit Insulinsekretagoga haben indirekt durch die Hypoglykämie-induzierten Stürze ein erhöhtes Risiko. Ein weiterer Grund dürfte die längere Krankheitsdauer (> 5 Jahre) und/oder eine schlechte glykämische Kontrolle zusammen mit sekundären Komplikationen (Retinopathie, Neuropathie, Nephropathie etc.) sein [22].

#### Inkretin-Mimetika

Beide Inkretin-Mimetika Dipeptidyl-Peptidase-4(DPP4)-Inhibitoren und Glucagon-like-peptide-1(GLP-1)-Analoga haben beim T2DM ein mehrheitlich neutrales skeletales Sicherheitsprofil. Der wahrscheinlichste Mechanismus ist der neutrale bis positive Effekt von GLP‑1 auf die Knochenformation durch Änderung der miRNA-Expression und das geringe Risiko in Bezug auf iatrogene Hypoglykämie [23].

#### SGLT-2-Inhibitoren

Rezente Daten über den Natrium-Glukose-Cotransporter 2(SGLT-2)-Inhibitor Canagliflozin beschreiben einen negativen Effekt im Sinne einer Abnahme der Knochenmineraldichte („bone mineral density“ [BMD]) und einer Zunahme des Knochenbruchrisikos. Der zugrunde liegende Mechanismus ist noch nicht vollständig entschlüsselt, aber SGLT-2-Inhibitoren hemmen im proximalen Tubulus die Reabsorption von Glukose und führen gleichzeitig zu einer vermehrten Reabsorption von Phosphat. Dies führt konsekutiv zu erhöhten Spiegeln von Serumphosphat, was ein möglicher Trigger für Parathormon (PTH) und einen erhöhten Knochenstoffwechsel ist.

Konträr zu Canagliflozin gibt es derzeit keinen Hinweis auf einen negativen Einfluss von Empagliflozin oder Dapagliflozin auf das Organsystem Knochen, weshalb den beiden letztgenannten Substanzen der Vorzug in dieser Substanzklasse gegeben werden sollte. Allerdings konnte eine Metaanalyse von 20 Studien mit Dapagliflozin, Empagliflozin oder Canagliflozin keine eindeutige Erhöhung der Frakturraten nachweisen. Aus derzeitiger Sicht ist bei Patienten mit T2DM Canagliflozin möglicherweise das Frakturrisiko erhöht [24].

## Diagnostik

### DXA-Knochendichtemessung

Die Knochendichtemessung mittels DXA („dual energy x‑ray absorptiometry“) ist nach wie vor der Goldstandard. Die Definition einer Osteoporose von einem T‑Score ≤ −2,5 basiert auf einer Definition der WHO aus dem Jahr 1994 und definiert die Erkrankung, jedoch nicht die individuelle Interventionsschwelle [25].

Die Mehrzahl der Studien bei Patienten mit T1DM zeigen, dass die BMD bei dieser Patientenpopulation deutlich vermindert ist [26]. Aufgrund der meist vorherrschenden Adipositas als Risikofaktor bei T2DM wäre grundsätzlich davon auszugehen, dass ein hoher Body Mass Index (BMI) und eine hohe BMD positiv miteinander korrelieren. Daher haben Patienten mit einem T2DM in der Regel eine 5–10 % höhere BMD im Vergleich zur nichtdiabetischen gesunden Population. Die höhere BMD ist vor allem beim jüngeren männlichen Geschlecht vorherrschend – interessanterweise auch bei höheren HbA_1c_-Werten. Die höhere BMD ist vor allem am gewichtstragenden Knochen zu sehen, jedoch nicht am Radius.

**Abb. 1:**
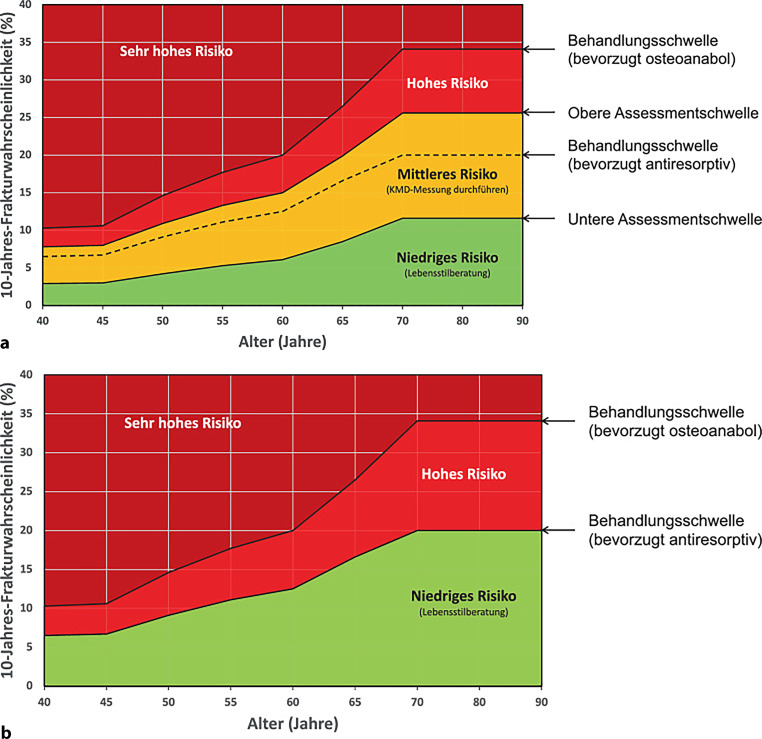
**a** Screening für osteoporotisches Frakturrisiko bei Personen ab 50 Jahren mittels FRAX®, aber noch *vor* einer Knochendichtemessung Alter (Jahre) 10-Jahres-Frakturwahrscheinlichkeit (%) Behandlungsschwelle (bevorzugt osteoanabol) Behandlungsschwelle (bevorzugt antiresorptiv) obere Assessmentschwelle untere Assessmentschwelle. (Adaptiert nach [45]). **b** Interventionsschwellen nach Risikoeinschätzung für eine „major osteoporotic fracture“ (*MOF*) bei Personen ab 40 Jahren mit FRAX® *und* Knochendichtemessung am Schenkelhals. [45]

Die relativ höhere BMD bei T2DM schützt die Patienten jedoch nicht vor Frakturen. Die Mehrzahl der Patienten mit Frakturen haben einen T‑Score im osteopenen Bereich, also einen T‑Score > −2,5 [27]. Bei Frauen mit T2DM ist das individuelle Frakturrisiko im Gegensatz zu Frauen ohne Diabetes in etwa 0,5 T-Scores als Korrekturfaktor tiefer als der tatsächliche Messwert anzusetzen (Abb. 1). Obwohl zahlreiche Studien in dieser Patientenpopulation bestätigen, dass die DXA-Messung systematisch das Frakturrisiko unterschätzt, werden unter Berücksichtigung dieses Korrekturfaktors vor allem ältere Patientinnen und Patienten adäquat stratifiziert [28].

**Abb. 2:**
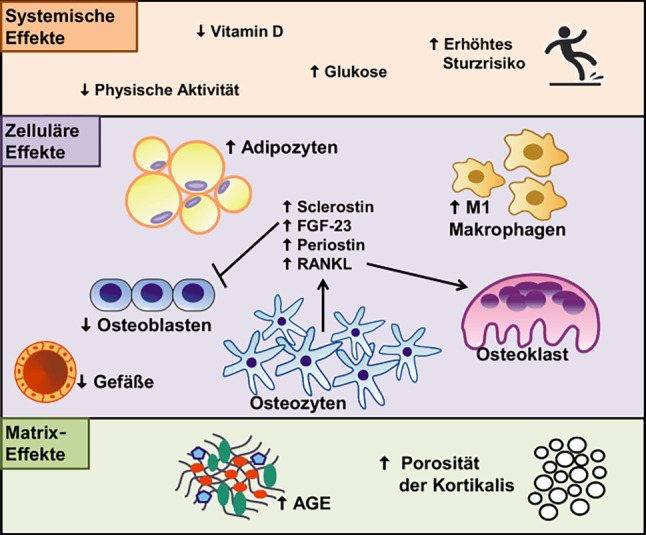
Effekte des T2DM auf den Knochenstoffwechsel. Auf systemischer Ebene ist T2DM mit einem niedrigen Vitamin-D-Spiegel, hohen Glukosewerten sowie eingeschränkter physischer Aktivität und einem erhöhten Sturzrisiko verbunden, alle, welche zu einer erhöhten Frakturrate führen. Auf zellulärer Ebene ist vor allem die Anzahl der Adipozyten erhöht, wobei die Anzahl der Osteoblasten sowie die Anzahl und Funktion der Gefäße vermindert sind. Osteozyten werden zur vermehrten Produktion von Sclerostin, Periostin, FGF-23 und RANKL animiert, welche die Osteoblastendifferenzierung hemmen und die Osteoklastengeneration fördern. Zusätzlich fördern proinflammatorische M1-Makrophagen die Osteoklastengeneration und hemmen Osteoblasten. T2DM hat auch direkte Effekte auf die Knochenmatrix. So werden vermehrt „advanced glycation endproducts“ (*AGE*) in die Kollagenmatrix eingebaut, die so zur Versteifung der Matrix beiträgt. Außerdem führt T2DM zur einer poröseren kortikalen Knochenstruktur, welches die mechanische Stabilität des Knochens beeinträchtigt

**Abb. 3:**
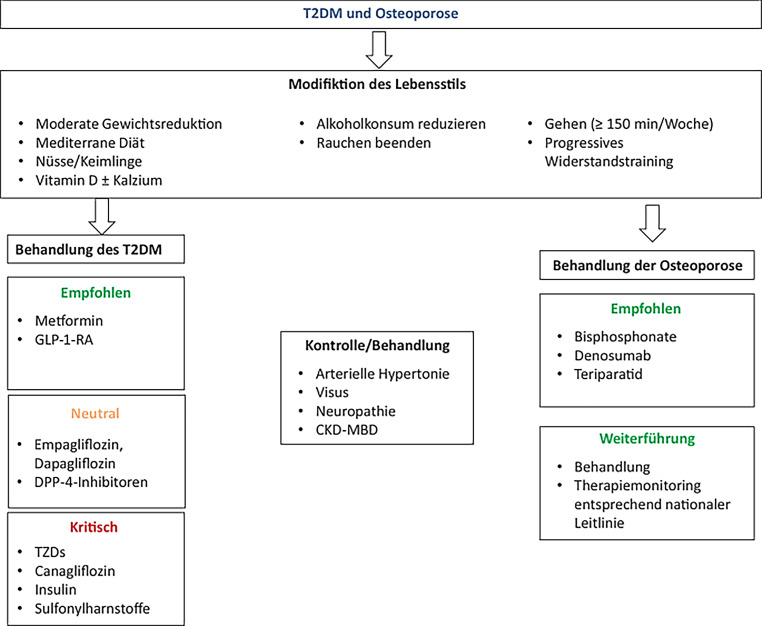
Strategien zur Behandlung von T2DM aus diabetologischer und osteologischer Sicht. *DPP4* Dipeptidyl-Peptidase-4-Inhibitor, *GLP‑1* Glucagon-like-peptide-1-Analoga, *TZD* Tiazolidindione, *SGLT2* Natrium-Glukose-Cotransporter 2 - Canagliflozin, *CKD-MBD* „chronic kidney disease – metabolic bone disease“. ^a^ Unter Berücksichtigung weiterer klinischer Merkmale entsprechend der ÖDG-LL zur antihyperglykämischen Therapie bei T2DM. (From: Diagnose und Management der Osteoporose bei Diabetes mellitus (Update 2019))

Manche Studien bestätigen einen schnelleren Verlust an BMD auch an gewichtstragenden Knochen (z. B. Hüfte) unter T2DM als möglichen Grund für die erhöhte Frakturrate [29].

Mit dem Trabecular Bone Score (TBS) steht eine Methode zur Verfügung, um aus einer zweidimensionalen DXA-Untersuchung Informationen über die Knochenmikrostruktur der Lendenwirbelsäule zu generieren. Diese einheitslose Zahl spiegelt anhand der Analyse von Grauwert-Variogrammen der radiologischen Messung der Lendenwirbelkörper L1–L4 mit einer hohen Korrelation die trabekuläre Mikroarchitektur bei Osteoporose unabhängig von der BMD wider [30]. Die TBS-Software kann direkt im Rahmen der Messung mit oder auch retrospektiv den Score berechnen, wodurch sich keine zusätzliche Strahlenbelastung für den Patienten ergibt. Im Gegensatz zur DXA-Methode ist der TBS bei Patienten mit T2DM tiefer als in einer nichtdiabetischen Population. In einer großen populationsbasierten Untersuchung wurden Patienten mit einem TBS von < 1230 als Risikopatienten für Osteoporose-assoziierte Frakturen eingestuft, bei einem TBS von 1230–1310 ein mittleres Risiko. Bei T2DM fanden sich in Studien TBS-Werte zwischen 1100 und 1200 [31].

Der TBS ist bei Patienten mit guter glykämischer Kontrolle höher und tiefer bei einem schlecht eingestellten T2DM. Der TBS ist somit ein unabhängiger Prädiktor für das Frakturrisiko bei Diabetes (HR 1,27) bzw. auch ohne Diabetes (HR 1,31) [32].

Alternative Methoden wie etwa der Ultraschall am Calcaneus oder am Radius zeigen inkonklusive Ergebnisse bei T2DM [33].

### Mikroarchitektur und Knochenqualität

Die BMD allein – vor allem beim T2DM – erklärt nicht die erhöhte skeletale Fragilität. Sowohl in MRT-Untersuchungen als auch mittels HR-pQCT („high resolution peripheral quantitative computed tomography“) am Radius (nicht gewichtstragender Knochen) und an der Tibia (gewichtstragender Knochen) zeigt sich beim T2DM eine verschlechterte Mikroarchitektur. Die Trabekel beim T2DM sind im Vergleich zum nichtdiabetischen Patienten eher hypertrophiert. Im trabekulären Netzwerk finden sich allerdings auch größere Löcher, zusätzlich ist die kortikale Porosität (bis zu 16 %) gegenüber Patienten ohne T2DM erhöht. Die strukturelle Alteration mit hoher Heterogenität ist an der endokortikalen Übergangszone besonders ausgeprägt („Trabekularisierung der Kortikalis“). Zusätzlich gibt es einen geschlechtsspezifischen Unterschied mit schlechteren Werten beim weiblichen Geschlecht [34, 35].

Aufgrund dieser strukturellen Defizite bei T2DM sind die Knochenfestigkeit sowie die Steifigkeit und die Elastizität des Knochens in virtuellen FEA(„finite element analysis“)-Untersuchungen vermindert. Microindentations-Untersuchungen zeigen zusätzlich strukturelle Einschränkungen als Ausdruck veränderter Kollagenverlinkungen in der Knochenmatrix aufgrund vermehrter AGEs („advanced glycation endproducts“). Diese Verlinkungen führen dazu, dass der Knochen an Elastizität und Flexibilität verliert. In Summe haben diese Untersuchungsergebnisse den Begriff der „Diabetoporose“ geprägt ([36]; Abb. 1a).

### Knochenstoffwechsel: Histomorphometrie und Serummarker

Der Goldstandard zur Untersuchung des lokalen Knochenstoffwechsels ist die Histomorphometrie aus bioptischen Proben. Zur Abschätzung der tatsächlichen Aktivität ist einer der wichtigen Parameter die Formationsrate bezogen auf eine Referenzoberfläche der Biopsie („bone formation rate/bone surface“ [BFR/BS]). Bei diabetischen Patientinnen und Patienten ist dieser Parameter im trabekulären, endokortikalen und intrakortikalen Bereich um bis zu 70–80 % vermindert [37].

In der Mehrzahl der Studien wurde bei Patienten mit T2DM eine verminderte Aktivität von serologischen Formationsmarkern („procollagen type I N‑terminal propeptide“ [PINP]; „osteocalcin“ [OC]) und Resorptionsmarkern („C telopeptide“ [CTX]; „tartrate-resistant acid phosphatase 5“ [TRAP5b]) nachgewiesen [14]. Der Zusammenhang zwischen dem „low bone turnover“ und der gleichzeitig nachgewiesenen strukturellen Alteration (kortikale Porosität) ist zum gegenwärtigen Zeitpunkt unklar.

### Alternative (experimentelle) biochemische Marker der Knochenfragilität

Bei Diabetes ist in der Knochenmatrix der Gehalt von Pentosidin, dem am häufigsten vorhandenen AGE, im Vergleich zu nichtdiabetischen Menschen deutlich erhöht. Erhöhte Pentosidin-Spiegel im Knochen und im Serum korrelieren negativ mit der biomechanischen Stärke des Knochens. Untersuchungen bestätigen erhöhte Werte von AGEs und auch von sRAGE („soluble receptors for advanced glycation endproducts“) als prädiktiven Faktor für eine erhöhte Inzidenz von klinischen und vertebralen Frakturen unabhängig von der BMD [38].

Sclerostin, der endogene Inhibitor des Wnt/β-Catenin-Signalweges und somit der Knochenformation durch Osteoblasten, ist bei T2DM deutlich erhöht. Die Höhe der Sclerostin-Spiegel korreliert bei diesen Patienten mit der Inzidenz von Fragilitätsfrakturen [39]. Bei T1DM verhalten sich die Sclerostin-Spiegel genau gegenläufig zur Inzidenz von Frakturen. Patienten mit obersten Drittel der gemessenen Spiegel hatten ein um 81 % geringeres Frakturrisiko verglichen mit Patienten mit Spiegeln im untersten Drittel [40]. Ob nun eine Erhöhung zirkulierender Sclerostin-Spiegel direkt die Dysfunktion von Osteozyten widerspiegelt und/oder ein Marker für eine zusätzliche Angiopathie ist, bleibt derzeit noch unbeantwortet [41].

Serum-Periostin bzw. dessen Fragmente sind mit einem erhöhten Frakturrisiko bei nichtdiabetischen Patienten vergesellschaftet. Derzeit laufen Studien in großen diabetischen Populationen zur Evaluation dieses Markers [42]. Die Bestimmung von Serum-microRNA(miRNA)-Signaturen erscheint nicht nur bei diabetischen Populationen zukünftig eine entsprechende Option zu werden ([43, 44]; Abb. 1a).

## Basisprophylaxe mit Vitamin D und Kalzium

Die Kalzium- und Vitamin-D-Substitution ist sowohl eine eigenständige Therapiemöglichkeit der Osteoporose als auch die absolut notwendige Basis jeder spezifischen Osteoporosetherapie.

Eine ausreichende Versorgung mit Vitamin D ist eine wichtige Voraussetzung für die Knochengesundheit. Eine 25-OH-Vitamin-D-Serumkonzentration < 20 ng/ml (50 nmol/l) ist mit einem erhöhten Risiko für proximale Femurfrakturen und nichtvertebrale Frakturen verbunden.

Zur Therapie eingesetzt wird Cholecalciferol (Vitamin D_3_); 1 μg Vitamin D_3_ entspricht 40 IE Vitamin D_3_. Die Einnahme soll mit den Mahlzeiten erfolgen, da dies die Resorption verbessert. Die Tagesdosis (z. B. 800 IE) kann auch als Wochenäquivalent gegeben werden (5600 IE einmal wöchentlich). Im Einzelfall kann bei Malabsorption eine parenterale (intramuskuläre) Gabe von 100.000 IE Cholecalciferol notwendig sein. Die Gabe der aktiven Form von Vitamin D – Calcitriol (1,25-Dihydroxycholecalciferol) – ist nur bei schwerer Niereninsuffizienz indiziert.

Eine ausreichende Kalziumzufuhr ist primär über die Nahrung sicherzustellen. Patientinnen und Patienten mit Osteoporose (mit und ohne spezifische Osteoporosetherapie) sollen daher täglich 1000 mg Kalzium aufnehmen, vorzugsweise über die Nahrung. Ist dies nicht möglich, sind Kalziumsupplemente erforderlich. Pro Einnahme wird eine Dosis von maximal 500 mg Kalziumsupplement empfohlen [25].

## Spezifische Osteoporosetherapie bei Diabetes

Keine einzige randomisierte Studie hatte bisher als Endpunkt die Wirksamkeit einer spezifischen Osteoporosetherapie bei Patienten mit T2DM. Daher basieren die Empfehlungen für das Management von Patienten mit Diabetes und einem erhöhten Frakturrisiko auf empirischen Daten und der klinischen Erfahrung. Die klinische Evidenz in Bezug auf die Effizienz einer antiresorptiven oder anabolen Osteoporosetherapie bei gleichzeitigem Diabetes beruht daher auf Post-hoc-Analysen von Subgruppen in großen randomisierten Osteoporosestudien und auch einer kleinen Anzahl von Observationsstudien [45].

Grundsätzlich sind sämtliche Medikamente zur Behandlung der Osteoporose auch bei Patientinnen und Patienten mit einem manifesten Diabetes möglich und zugelassen. Da sowohl der Diabetes mellitus als auch die Osteoporose eine chronische Erkrankung mit einem dauerhaft erhöhten Risiko für sekundäre Komplikationen sind, ist die Indikation für eine langfristige Behandlung indiziert.

### Bisphosphonate

Bisphosphonate (Alendronat, Risedronat, Ibandronat, Zoledronat) sind potente Inhibitoren der Knochenresorption. Sie werden an metabolisch aktiven Umbaueinheiten im Knochen abgelagert und bewirken eine Apoptose von Osteoklasten. Die Resorptionsaktivität wird im Gesamtskelett deutlich gedämpft und das Frakturrisiko reduziert.

Oral werden Bisphosphonate nur in geringem Ausmaß (maximal 3 %) resorbiert; die Einnahme erfolgt stets nüchtern in ausreichendem Abstand zur Nahrungsaufnahme, mit ausreichend Wasser und in aufrechter Körperhaltung, um Irritationen der Ösophagusschleimhaut zu vermeiden.

Bei intravenöser Bisphosphonat-Gabe kann, überwiegend bei erstmaliger Verabreichung, eine sog. „Akutphasereaktion“ – im Wesentlichen ein grippeähnliches Zustandsbild mit Fieber und Muskelschmerzen – auftreten, die in der Regel innerhalb von 36 h nach intravenöser Gabe beginnt und dann 24–48 h anhält.

Bei allen Bisphosphonaten stellen die Hypokalzämie, eine erhebliche Nierenfunktionseinschränkung oder eine Gravidität eine Kontraindikation dar.

Bisphosphonate haben eine lange Verweildauer im Knochen. Residuale Wirkungen auf den Knochenstoffwechsel lassen sich auch nach Beendigung der Bisphosphonat-Therapie nachweisen. Das Auftreten von atypischen Femurfrakturen ist sehr selten, scheint aber unter einer Langzeitgabe mit Bisphosphonaten zuzunehmen. Kiefernekrosen sind bei dieser für Osteoporose zugelassenen Therapie eine mutmaßlich seltene Nebenwirkung. Eine Kontrolle des Zahnstatus ist allerdings vor Therapiebeginn empfehlenswert.

Es gibt keine durch Frakturdaten validierten individuellen Entscheidungskriterien für die Wiederaufnahme einer Therapie nach einer Therapiepause oder einen weiteren Therapieverzicht in Abhängigkeit von Veränderungen der BMD, der Knochenumbaumarker oder anderer messtechnischer oder klinischer Kriterien. Datenbankanalysen geben allerdings Hinweise auf einen Wiederanstieg des Knochenbruchrisikos nach Absetzen einer Bisphosphonat-Therapie [25].

### Denosumab

Denosumab ist ein monoklonaler Antikörper gegen RANKL, der die Reifung und Aktivierung der Osteoklasten hemmt. Es wird alle 6 Monate subkutan verabreicht und wird nicht renal eliminiert.

Bei der Behandlung der postmenopausalen Osteoporose ist eine Reduktion von vertebralen und nichtvertebralen Frakturen inklusive proximaler Femurfrakturen in Studien bis zu 10 Jahre nachgewiesen. Die Wirkung ist unabhängig von einer eventuellen Vorbehandlung mit Bisphosphonaten [46]. Die Behandlungsdauer ist unklar. Nach Absetzen von Denosumab scheint es im Gegensatz zu den Bisphosphonaten zu einem raschen Anstieg des Knochenumbaus und in weiterer Folge zu einer Abnahme der Knochenmineraldichte zu kommen. Kiefernekrosen und atypische Femurfrakturen sind bei dieser für Osteoporose zugelassenen Therapie eine mutmaßlich sehr seltene Nebenwirkung [25].

### Raloxifen

Raloxifen ist ein selektiver Östrogenrezeptormodulator (SERM), der die Knochenresorption hemmt und das Frakturrisiko für vertebrale Frakturen reduziert (nicht für nichtvertebrale Frakturen und proximale Femurfrakturen). Raloxifen ist zugelassen für die Prävention und für die Therapie der Osteoporose bei postmenopausalen Frauen.

Ein bedeutender zusätzlicher Effekt ist die Reduktion des relativen Risikos eines invasiven (Östrogenrezeptor-positiven) Mammakarzinoms um 79 %. Eine unerwünschte Nebenwirkung ist die Erhöhung des thromboembolischen Risikos [25].

### Teriparatid

Teriparatid, ein aminoterminales Fragment des Parathormons, wird einmal täglich subkutan über 24 Monate angewandt. Der osteoanabole Effekt beruht auf einer Beschleunigung der Reifung und Stimulierung von Osteoblasten.

Im Anschluss an die anabole Reaktion des Knochens kommt es nach Beendigung der Teriparatid-Therapie wiederum zu einem gesteigerten Knochenabbau, weshalb eine sofortige Anschlussbehandlung mit einem Antiresorptivum (Bisphosphonat, Denosumab, SERM) unbedingt notwendig ist ([25]; Tab. 2).

**Tab. 2:** Effekte spezifischer Osteoporosemedikamente bei Patienten mit einem T2DM auf BMD und Frakturrisiko [45]

Spezifisches Osteoporosemedikament	BMD (T2DM)	Frakturrisiko in Bezug auf T2DM
Alendronat	↑	KD/↔
Risedronat	↑	KD
Ibandronat	KD	KD
Zoledronat	KD	KD
Raloxifen	KD	↓/↔
Denosumab	KD	KD
Teriparatid	↑	↔
Romosozumab	↑	KD

↑ Verbesserung/Erhöhung, ↓ Verminderung, ↔ unverändert

*KD* keine Daten

### Romosozumab

Romosozumab ist ein humanisierter monoklonaler Antikörper, der Sclerostin hemmt. Dadurch fördert er die Knochenbildung über Osteoblasten und hemmt gleichzeitig die Knochenresorption durch Osteoklasten. Zugelassen ist Romosozumab seit Oktober 2019 für postmenopausale Frauen mit schwerer Osteoporose und sehr hohem Frakturrisiko. Eine Zulassung für Männer liegt derzeit nicht vor.

Die empfohlene Dosis beträgt 210 mg (2 subkutane Injektionen à 105 mg) einmal monatlich über maximal 12 Monate. Im Anschluss ist eine Folgetherapie mit einem antiresorptiven Medikament erforderlich, um eine Behandlungslücke zu vermeiden.

In klinischen Studien zeigte Romosozumab eine signifikante Reduktion neuer Wirbelkörper- sowie klinischer Frakturen im Vergleich zu Placebo nach 12 Monaten. Auch nach einer anschließenden Denosumab-Therapie blieb dieser Vorteil bestehen. Unerwünschte Ereignisse einschließlich schwerwiegender kardiovaskulärer Ereignisse traten in ähnlicher Häufigkeit auf.

Im Vergleich zu Teriparatid zeigte sich unter Romosozumab eine stärkere Zunahme der Knochendichte (KMD), insbesondere an der Hüfte. Vergleichsdaten zur Frakturreduktion liegen hierfür jedoch nicht vor.

In einer aktiv kontrollierten Studie war eine sequenzielle Therapie mit Romosozumab gefolgt von Alendronat wirksamer in der Frakturreduktion als eine alleinige Alendronat-Therapie. Allerdings traten in der Romosozumab-Gruppe während des ersten Behandlungsjahres häufiger kardiovaskuläre Ereignisse auf.

Romosozumab ist kontraindiziert bei Hypokalziämie, Überempfindlichkeit gegen Bestandteile des Präparats sowie bei anamnestischem Myokardinfarkt oder Schlaganfall. Bei über 400.000 behandelten Patientinnen wurden keine neuen Sicherheitsbedenken identifiziert; die Inzidenz kardiovaskulärer Ereignisse blieb stabil.

Vor Therapiebeginn müssen das Fraktur- und das kardiovaskuläre Risiko sorgfältig abgewogen werden. Hypokalziämie sollte zuvor korrigiert werden, eine adäquate Versorgung mit Kalzium und Vitamin D ist sicherzustellen. Bei Patientinnen mit schwerer Niereninsuffizienz oder unter Dialyse ist das Risiko einer Hypokalziämie erhöht. Sehr selten wurden Osteonekrosen des Kiefers und atypische Femurfrakturen berichtet [47, 48].

### Abaloparatid

Abaloparatid ist bereits in der EU zugelassen, jedoch noch nicht in Österreich verfügbar.

## Management einer erhöhten Knochenfragilität bei Diabetes

Die Kriterien für den Beginn einer osteologischen Therapie bei Diabetes basieren entweder auf einer prävalenten Fragilitätsfraktur (unabhängig von der BMD) und/oder auf einer verminderten BMD. Das diagnostische Kriterium der Osteoporose in der DXA-Messung (T-Score < 2,5) ist nicht mit der individuellen Therapieschwelle gleichzusetzen [25, 49].

Die wichtigste Entscheidungshilfe für den Beginn einer Therapie ist auch beim Patienten mit Diabetes eine prävalente Fragilitätsfraktur. Das Ziel ist jedoch, die Patienten vor der ersten niedrig-traumatischen Fraktur zu schützen.

### BMD-Interventionsschwelle

Bei Patienten mit einem manifesten T2DM unterschätzt die DXA-Messung das individuelle Frakturrisiko. Aktuell wird daher bei diesen Patienten eine Anhebung der Interventionsschwelle auf einen T‑Score von −2,0 an der Lendenwirbelsäule (kumulativ L1–L4) oder an der Hüfte (Schenkelhals bzw. gesamte Hüfte) empfohlen, um der DXA-basierten Unterschätzung der Knochenfragilität entgegenzuwirken. Diese Anhebung ist jedoch nur in westlichen Populationen zu empfehlen. Patienten aus Asien oder dem Nahen/Mittleren Osten haben Alters- und Geschlechts-adjustieren BMD niedrigere Frakturraten, die sich auch bei manifestem Diabetes auswirken.

Patienten mit einem ausgeprägten Verlust an BMD in 2 konsekutiven Messungen (= > 5 % in 2 Jahren) sollte schon bei Werten nahe der Interventionsschwelle prophylaktisch behandelt werden.

### FRAX®

FRAX, das WHO zertifizierte Fracture Risk Assessment Tool, implementiert nationale Frakturdaten und besteht aus 12 dichotomisierten Fragen (die 12. Frage zu BMD ist optional) [50]. Dem FRAX liegen klinische Risikofaktoren („clinical risk factor“ [CRF]) zugrunde, die in randomisierten Studien ein erhöhtes individuelles Risiko für Fragilitätsfrakturen sind. FRAX berechnet 2 Werte: (a) eine 10-Jahres Wahrscheinlichkeit für alle osteoporotischen Frakturen und (b) eine 10-Jahres Wahrscheinlichkeit für eine osteoporotische Hüftfraktur. Entsprechend der österreichischen Leitlinie mit Erscheinungsdatum Herbst 2024 zur Behandlung der Osteoporose werden altersdynamisierte Behandlungsschwellen in Halbdekaden für Frauen und Männer empfohlen.

Diabetes per se ist im FRAX kein eigener CRF, daher unterschätzt auch dieses diagnostische System die Frakturwahrscheinlichkeit bei einem manifesten Diabetes. Diabetes per se ist allerdings ein starker Risikofaktor für eine osteoporotische Fraktur, auch nach Korrektur aller CRFs und BMD [51]. FRAX bietet auch die Möglichkeit, die BMD-Werte mittels TBS-Korrektur zu rechnen. Vor allem bei T2DM führt dies zu einer Verbesserung der Vorhersagewahrscheinlichkeit.

Für die Ersteinschätzung der 10-Jahres-Frakturwahrscheinlichkeit einer hüftnahen Fraktur und/oder einer MOF bei Personen ab 50 Jahren vor Durchführung einer Knochendichtemessung steht eine auf die österreichische Bevölkerung kalibrierte Version des FRAX® zur Verfügung (https://frax.shef.ac.uk/FRAX/tool.aspx?lang=de). Lebensalter (x-Achse) und die 10-Jahres-Frakturwahrscheinlichkeit im FRAX® (y-Achse) ergeben die Zuordnung zu einer der Risikokategorien. Da Diabetes mellitus ein CRF ist, wird daher ein Screening für diese Patientenpopulation empfohlen.

## Aus 4 Farben werden 3 Farben

Bei einer 10-Jahres-Frakturwahrscheinlichkeit im grünen Bereich sind keine weiteren Maßnahmen erforderlich. Bei einer hohen 10-Jahres-Frakturwahrscheinlichkeit (= roter Bereich) wird eine antiresorptive und bei einem sehr hohen Risiko (= dunkelroter Bereich) eine osteoanabole Therapie empfohlen.

Fällt die ermittelte 10-Jahres-Frakturwahrscheinlichkeit in die mittlere (= gelbe) Risikokategorie, sollte eine Knochendichtemessung durchgeführt und das Ergebnis der DXA-Messung am Schenkelhals für eine neuerliche Berechnung in das FRAX®-Tool integriert werden. Nach Integration dieser Knochendichtemessung fällt bei der Neuberechnung die mittlere (= gelbe) Risikokategorie weg → aus 4 Farben werden 3 Farben = Behandlungskategorien (Basismaßnahmen, antiresorptive oder osteoanabole Therapie) (Abb. 1; [45]).

**Abb. 4:**
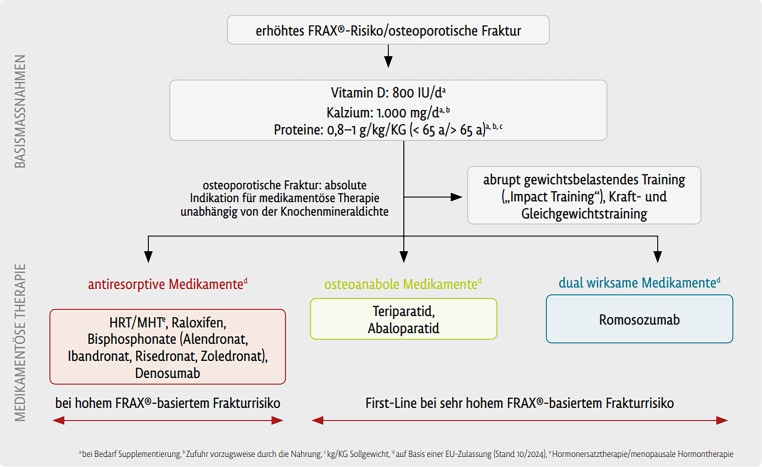
Therapiealgorithmus mit den Basismaßnahmen und den medikamentösen Empfehlungen entsprechend dem FRAX-basierten Risiko. *HRT* Hormonersatztherapie, *MHT* menopausale Hormontherapie

Mit Verweis auf das Update der Österreichischen Osteoporose Leitlinie ergibt sich somit folgender Therapiealgorithmus für Basismaßnahmen und spezifische medikamentöse Therapie ([45]; Abb. 4; Tab. 3).

**Tab. 3:** Risikofaktoren für Frakturen bei Patienten mit Diabetes

*Allgemeine Risikofaktoren*	FRAX® CRF: Alter, Geschlecht, prävalente Fragilitätsfrakturen, Hüftfraktur der Mutter oder des Vaters, gegenwärtiges Rauchen, Alkohol (≥ 3 Einheiten/Tag), Glukokortikoide, rheumatoide Arthritis, BMD-Schenkelhals
*Krankheitsspezifische Risikofaktoren*	Dauer des Diabetes > 5 Jahre
	Antidiabetogene Medikamente: Insulin, TZDs, Canagliflozin, Sulfonylharnstoffe
	HbA_1c_ > 7 %
	Mikrovaskuläre Komplikationen: periphere und autonome Neuropathie, Retinopathie, Nephropathie

*FRAX* Fracture Risk Assessment Tool der WHO, *CRF* „clinical risk factors“, *TZD* Thiazolidindione, *SGLT2* Natrium-Glukose-Cotransporter 2

## Fazit

Patienten mit Diabetes haben ein erhöhtes Risiko für Fragilitätsfrakturen. Die Pathophysiologie ist unklar und vermutlich multifaktoriell. Longitudinale Studien haben den Nachweis erbracht, dass FRAX und BMD mittels DXA(T-Score)-Messungen und eines eventuell vorhandenen TBS das individuelle Frakturrisiko vorhersagen können. Hierfür muss allerdings eine Adjustierung vorgenommen werden, um das Risiko nicht zu unterschätzen.

Bei der Wahl der antihyperglykämischen Therapie sollen Substanzen mit nachgewiesenem negativem Effekt auf den Knochen nicht zum Einsatz kommen. Bei Vorliegen einer Fragilitätsfraktur ist auf jeden Fall – unabhängig von allen vorliegenden Befunden – eine langfristige spezifische osteologische Therapie indiziert.

Es gibt zurzeit keine Daten aus einem rein diabetischen Kollektiv, weshalb der hier empfohlene Algorithmus einen Konsens einer internationalen Expertengruppe (somit Evidenzgrad D) darstellt.

Demnach sollen Patienten mit Diabetes mellitus und einem erhöhten Frakturrisiko wie Patienten ohne Diabetes behandelt werden. Auf einen optimalen Vitamin-D-Spiegel sowie eine ausreichende Kalziumaufnahme (vorzugsweise durch die Nahrung) sollte geachtet werden.

Zur Prävention von Fragilitätsfrakturen sind antiresorptive Medikamente die erste Wahl, entsprechend den nationalen Erstattungskriterien auch anabole Medikamente. Das Therapiemonitoring soll im Einklang mit der nationalen Osteoporose-Leitlinie erfolgen [45].

## Interessenkonflikt

C. Muschitz hat von folgenden Firmen, die auch fördernde Mitglieder der ÖGKM (Österr. Gesellschaft für Knochen- und Mineralstoffwechsel) und der ÖDG sind, Honorare für Vorträge und Beratung sowie wissenschaftliche Grants erhalten: Amgen Österreich; Eli Lilly Österreich, Novartis Pharma Österreich. Es besteht kein Konflikt mit der Erstellung dieses Manuskripts. A. Kautzky-Willer hat von folgenden Firmen, die teils auch fördernde Mitglieder der ÖDG sind, Honorare für Vorträge und Beratung erhalten: AstraZeneca, Amgen, Boehringer Ingelheim, Eli Lilly, Merck Sharp & Dohme, Novartis, Novo Nordisk, Roche, Sanofi-Aventis, Servier, Takeda. M. Rauner hat von folgenden Firmen, die auch fördernde Mitglieder der ÖGKM sind, Honorare für Vorträge und Beratung erhalten: Amgen. Es besteht kein Konflikt mit der Erstellung dieses Manuskripts. Y. Winhöfer-Stöckl hat von folgenden Firmen, die auch teils auch fördernde Mitglieder der ÖDG sind, Honorare für Vorträge und Beratung erhalten: Amgen, AstraZeneca, Eli Lilly, Novartis, Sanofi. J. Haschka hat keine Honorare von Firmen für Vorträge oder Beratung erhalten.
